# Safety and Efficacy of Stereotactic Body Radiation Therapy in the Treatment of Pulmonary Metastases from High Grade Sarcoma

**DOI:** 10.1155/2013/360214

**Published:** 2013-10-01

**Authors:** Niraj Mehta, Michael Selch, Pin-Chieh Wang, Noah Federman, Jay M. Lee, Fritz C. Eilber, Bartosz Chmielowski, Nzhde Agazaryan, Michael Steinberg, Percy Lee

**Affiliations:** ^1^Department of Radiation Oncology, David Geffen School of Medicine at UCLA, 200 UCLA Medical Plaza, B265, Los Angeles, CA 90095, USA; ^2^UCLA Jonsson Comprehensive Cancer Center, 10833 Le Conte Ave, Los Angeles, CA 90095, USA; ^3^Division of Pediatric Hematology Oncology, Department of Pediatrics, David Geffen School of Medicine at UCLA, Los Angeles, CA 90095, USA; ^4^Division of Thoracic Surgery, Department of Surgery, David Geffen School of Medicine at UCLA, Los Angeles, CA 90095, USA; ^5^Division of Surgical Oncology, Department of Surgery, David Geffen School of Medicine at UCLA, Los Angeles, CA 90095, USA; ^6^Division of Hematology Oncology, Department of Medicine, David Geffen School of Medicine at UCLA, Los Angeles, CA 90095, USA

## Abstract

*Introduction*. Patients with high-grade sarcoma (HGS) frequently develop metastatic disease thus limiting their long-term survival. Lung metastases (LM) have historically been treated with surgical resection (metastasectomy). A potential alternative for controlling LM could be stereotactic body radiation therapy (SBRT). We evaluated the outcomes from our institutional experience utilizing SBRT. *Methods*. Sixteen consecutive patients with LM from HGS were treated with SBRT between 2009 and 2011. Routine radiographic and clinical follow-up was performed. Local failure was defined as CT progression on 2 consecutive scans or growth after initial shrinkage. Radiation pneumonitis and radiation esophagitis were scored using Common Toxicity Criteria (CTC) version 3.0. *Results*. All 16 patients received chemotherapy, and a subset (38%) also underwent prior pulmonary metastasectomy. Median patient age was 56 (12–85), and median follow-up time was 20 months (range 3–43). A total of 25 lesions were treated and evaluable for this analysis. Most common histologies were leiomyosarcoma (28%), synovial sarcoma (20%), and osteosarcoma (16%). Median SBRT prescription dose was 54 Gy (36–54) in 3-4 fractions. At 43 months, local control was 94%. No patient experienced G2-4 radiation pneumonitis, and no patient experienced radiation esophagitis. *Conclusions*. Our retrospective experience suggests that SBRT for LM from HGS provides excellent local control and minimal toxicity.

## 1. Introduction

Although sarcomas are rare, accounting for less than 1% of cancers in adults, 20–40% of all sarcoma patients ultimately develop metastatic disease in the lungs [1–4]. Of the high-grade sarcomas (HGS), 40–60% of patients will develop lung metastases (LM), of which 70–80% will have disease limited to the lungs, likely through hematogenous spread. The development of lung metastatic disease is associated with poor outcomes as few patients achieve durable disease control [5]. Many sarcomas have a unique biological predilection for the lung, often the only signs of metastatic disease, and, therefore, controlling these specific sites of progression can increase survival.

Due to this predilection of HGS for the lungs, surgical resection has been a vital part of managing these patients. Reported 5-year overall survival rates following metastasectomy vary from 20 to 40% depending upon the patient selection [5–10]. Over the past two decades, many groups have published results demonstrating successful palliation and even prolongation of survival after resection of LM from HGS [11]. Nearly 80% of the patients, however, will experience recurrent pulmonary disease even after a complete oncologic resection [8, 12–16].

Radiation therapy is another local modality that has been used for treating metastatic disease in the chest. Traditionally, radiation has had a limited, typically palliative role in the management of LM due to limited radiation tolerance of the lung. Achievements in radiation technique, dose delivery, image guidance, and precision have expanded indications for RT in the management of pulmonary disease.

With the development of stereotactic body radiation therapy (SBRT), high biologic doses of radiation using a precise arrangement of beams to target the tumor plus a small margin can be delivered, minimizing dose to the surrounding normal tissue. In primary nonsmall cell lung cancer, SBRT is becoming a standard therapeutic option for medically inoperable patients. The Radiation Therapy Oncology Group (RTOG) phase II trial reported a 3-year local control 97.6% with 54 Gy in 3 fractions [17]. Retrospective data have demonstrated equivalently high and durable local control using SBRT, approaching that of lobar resection [18]. Several institutions have utilized SBRT for the treatment of pulmonary oligometastatic disease [19, 20]. Investigators at the University of Colorado recently reported a 96% local control rate in 38 patients with 63 lung lesions in phase II trial [20].

At our institution, we also have treated a cohort of patients with metastatic HGS in the lungs with SBRT after a consensus recommendation by our multidisciplinary tumor board. Based on our results with treating early-stage NSCLC, we hypothesized that SBRT would be an efficacious approach for patients with LM from HGS. 

## 2. Materials and Methods

From March 2009 to October 2011 we treated a cohort of 16 consecutive patients with LM from HGS with linear accelerator-based SBRT at the University of California, Los Angeles, Department of Radiation Oncology. The institutional review board approved this study. Cases suitable for SBRT were selected after multidisciplinary evaluation at thoracic tumor board based upon high operative risk, refusal of surgery, documented metastatic disease outside of the lung, prior thoracotomy, and/or inability to tolerate lobectomy. Upon the first diagnosis of LM from HGS, patient and tumor characteristics were gathered, and then evaluation for surgical resection, chemotherapy, or potentially SBRT was completed. All patients did not receive surgical therapy but did receive some sort of systemic chemotherapy depending on the diagnosis. The lung had not received any sort of conventional radiation prior to the delivery of SBRT.

### 2.1. Simulation/Contours/Treatment

During simulation, all patients were immobilized with a vacuum bag in the supine arms-up position and underwent four-dimensional CT simulation (4D CT), obtaining 2.5 mm slices with free-breathing approach. The 4D CT generated an actual volumetric spatiotemporal anatomical data set by binning corresponding images from all couch positions into different volumes. Ultimately, 4 separate CT data sets were generated: original contrast CT simulation scan, end-expiratory, 50% expiration, and end-inspiratory for target volume definition.

To account for tumor motion, an internal target volume (ITV) was generated by contouring the gross tumor volume (GTV) on each of the four volume sets defined as tumor seen on lung windows. To create the final planning target volume (PTV), 0.3 cm margins were added in the lateral, anterior, and posterior dimensions, while 0.6 cm margins were added in the superior-inferior margins, making adjustments for other critical structures of avoidance such as the rib cage. The heart, lungs, esophagus, proximal tracheobronchial tree, spinal cord, and brachial plexus were contoured according to the guidelines detailed in RTOG 0236.

Treatment planning was performed using the iPLAN system (BrainLAB, AG, Heimstetten, Germany), prescribing to cover 95% of the PTV with the prescription dose delivered by multiple coplanar and noncoplanar conformal arcs or intensity modulated fields. Total dose and dose per fraction delivered were primarily 54 Gy in 3 fractions (18 Gy per fraction) or 50 Gy in 4 fractions (12.5 Gy per fraction) for more central lesions. A central lesion was defined as a tumor within 2 cm of the proximal bronchial tree (RTOG definition) [21]. Two patients received 36 Gy in 3 fractions and one patient received 42 Gy in 3 fractions, as they were part of an institutional combined SBRT and radiofrequency ablation (RFA) protocol. We adhered to the organ tolerance dose limits as specified by RTOG 0236 for peripheral tumors and with modified dose limits for a 4-fraction regimen. 

Stereoscopic and volumetric-based image-guidance were employed prior to each fraction. Respiratory gating was not employed. All patients were treated on the Novalis TX system, using Novalis ExacTrac patient positioning platform (BrainLAB, AG, Heimstetten, Germany). Patients received a cone beam CT scan prior to each fraction to ensure that the plan corresponded correctly to the patient's anatomy during treatment, and adjustments were made accordingly.

### 2.2. Chart Review

Routine follow-up included history and physical examination and a contrast-enhanced chest CT scan every 3–6 months after treatment. FDG-PET scans were only acquired if there was a concern for gross tumor recurrence in the background of an inflammatory lung reaction. All other inpatient/outpatient hospital notes, follow-up notes, and imaging were reviewed. In particular, we asked patients if they were experiencing fatigue, dyspnea, chest-wall pain, hemoptysis, or cough at their first follow-up visit at 3 months. The specific toxicities of radiation pneumonitis (RP) and radiation esophagitis (RE) were tracked longitudinally and scored on basis of Grade 0-1 (asymptomatic or no treatment) versus Grades 2–5 using the National Cancer Institute common toxicity criteria (CTC) version 3.0.

### 2.3. Statistical Analysis

The primary endpoint for this study was local control and the secondary endpoint was actuarial overall survival, estimated using Kaplan-Meier method. Gross tumor volume and the biologic equivalent dose (BED) were calculated for each individual lesion. The BED was calculated using the linear quadratic (LQ) formula:

(1) $$\begin{array}{c} \text{BED}=d\left[1+\frac{d/n}{\alpha /\beta }\right], \end{array}$$

assuming an *α*/*β* ratio of 10 for the tumor (*n* = number of fractions, *d* = total dose) [22]. Local failure was scored as an even if a treated lesion grew at any point on axial slices on 2 consecutive CT scans based on a radiographic evaluation or growth after initial shrinkage. 

Local control was defined as not having local failure at the time of the analysis (CT progression on two consecutive scans or tumor growth after initial shrinkage). Progression-free survival was defined as the time between the first day of SBRT to local failure, or last follow-up. Overall survival was defined as the time between the first day of SBRT to death, or last follow-up.

## 3. Results

Patient demographics and tumor characteristics are presented in Table 1. All patients received chemotherapy and a subset also underwent prior pulmonary metastasectomies (38%). The mean and median durations from the development of metastatic disease to lung SBRT were 19.6 and 13.7 months, respectively. One patient had RFA to a separate lesion prior to SBRT, while 2 patients had RFA to the radiated lesion within 7 days after SBRT on an institutional prospective protocol. Fifteen of 16 patients had chemotherapy prior to SBRT, and twelve of 16 patients had chemotherapy after SBRT. Five of 16 (31.3%) patients developed subsequent pulmonary recurrences after SBRT.

A total of 25 (76% soft tissue sarcoma, 24% bone sarcoma) lesions were treated and evaluable for this analysis (1–3 lesions treated at any given radiation session), of which 76% were noncentral lesions. Most common histologies were leiomyosarcoma (28%), synovial sarcoma (20%), and osteosarcoma (16%). Median patient age was 56 years (range: 12–85) and median follow-up time was 20 months (range: 3–43 months) for local control, and 25 months (range: 19–48 months) for overall survival. Median SBRT prescription dose was 54 Gy (range: 36–54 Gy) in 3-4 fractions (majority receiving 54 Gy in 3 fractions). Median PTV volume was 9.2 cm^3^ (range: 1.8–84.9 cm^3^).

**Figure 1:**
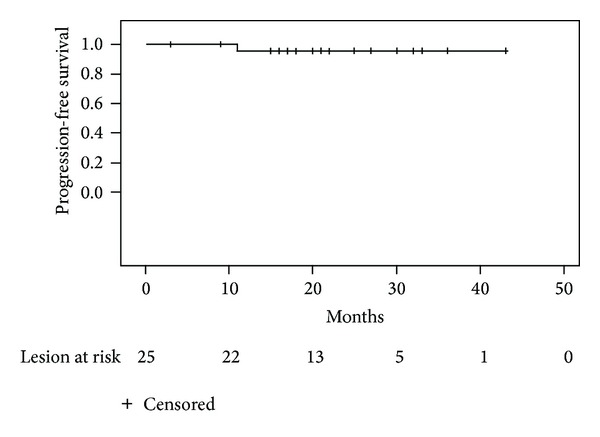
Local control. Actuarial local control estimated for the entire cohort using the Kaplan-Meier method.

At 43 months, there was one local failure (LC = 94%) in this cohort (Figure 1). The single failure occurred in a 72-year-old male, diagnosed with prostate leiomyosarcoma in 2008 and subsequent lung metastasis in the right upper lobe in 2010. The patient underwent 54 Gy in 3 fractions for an 11 by 10 mm lesion. SBRT was given with sequential gemcitabine chemotherapy. After 6 months, the lesion measured 6 by 3 mm. After 10 months from treatment, CT showed increase in the size of the lesion to 13 by 11 mm. But, because the lesion initially decreased in size and then demonstrated subsequent growth, we categorized this as a local failure. That lesion has since then remained unchanged. The patient has been treated with taxotere and doxorubicin and is currently on a clinical trial with pazopanib. He has since then developed multiple subcentimeter nodules in the lung that have been stable on the trial drug.

**Figure 2:**
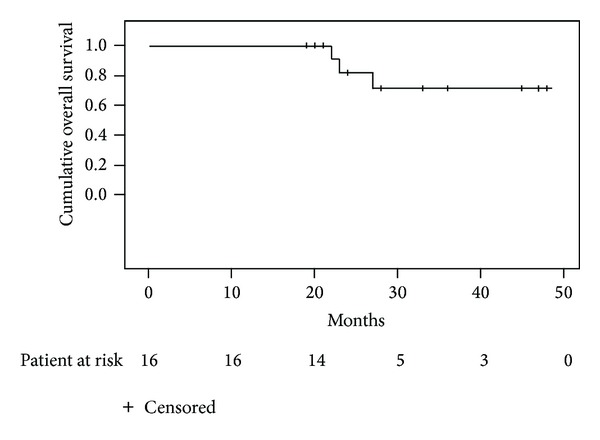
Overall survival. Actuarial overall survival estimated for the entire cohort using the Kaplan-Meier method.

Overall survival at 4 years was 72% (Figure 2). No patient experienced Grades 2–4 RP (Table 2), which are symptoms of shortness of breath requiring steroids and/or diuretics or worse. All but one patient had radiographic changes on the posttreatment CT, classified as Grade I RP. No patients experienced RE. No untoward late complications of the treatments were seen in these patients, although the follow-up time is limited.

## 4. Discussion

Metastatic disease historically portends a poor prognosis in cancer regardless of the histology of the primary. In particular, in HGS, patients with lung metastases unaddressed by surgical management have a median overall survival of 8–14 months [6, 8, 23]. Although there are no prospective randomized trials comparing pulmonary metastasectomy to observation, it is generally accepted that in patients with limited extrapulmonary metastatic disease, surgical resection of LM improves overall survival. Five-year survival rates in patients with LM from STS after surgical management range from 21 to 43% [1, 23]. Therefore, given the unique biology of many sarcomas, controlling sites of metastatic disease in the lung assuming the primary tumor site is managed appropriately can potentially increase long-term survival [6, 11]. 

Although primary surgical management remains the primary modality of treatment, our series supports the ability of SBRT to successfully control focal metastatic disease and extend survival in properly selected patients. Out of 25 lesions, there was 1 local progression and 78% survival at 4 years. In comparison, another recent dual-institution analysis treated 74 pulmonary lesions secondary to soft tissue sarcoma with SBRT using 50 Gy in 5 fractions, reporting a Kaplan-Meier estimate of 88% LC at 2 years and 82% at 3 years [24]. Taken together, SBRT is an attractive and promising alternative to surgery for oligometastatic disease to the lung from HGS. Given the excellent local control, and more importantly the overall survival in this series, SBRT may confer similar disease-specific and overall survival benefits to surgery. However, a prospective study is needed to validate these findings.

Most importantly, in our cohort, the management of LM with SBRT is associated with a favorable toxicity profile with the absence of symptomatic RP and RE. In one study, symptomatic grade 2–4 RP has been shown to be correlated with the mean lung dose and the V20 (volume of the lung receiving more than 20 Gy) [25], which can help to plan the radiation accordingly to minimize the chances of such subacute effects in the lung. Guckenberger et al. demonstrated that the onset of symptomatic pneumonitis is generally later than conventionally fractionated therapy, occurring in 10% of patients at a median interval of 5 months [26]. RTOG 0236 reported 12.7% Grade 3 and 3.6% Grade 4 protocol specified treatment-related adverse events. Since then, more attention has been paid to tailoring a dose according to the tumor volume, location, and proximity to central critical structures as well as skin, chest wall, and brachia plexus. Therefore, we can use prior studies of SBRT to determine therapeutic doses and potentially even use a BED calculation to come up with an effective yet safe dose to minimize the chance of pneumonitis, bronchial stenosis, and fistula [27, 28]. 

Furthermore, in selecting patients appropriate for SBRT, we can start with the vast literature providing prognostic variables that affect survival after surgery [1, 11, 14, 29]. Potential considerations include control of primary site of disease, other distant sites of metastases, disease-free interval prior to LM, probability of complete resection of all pulmonary lesions, location of lesions (laterality), histologic grade, amount of parenchyma needed to be removed, adequate cardiopulmonary reserve, and responsiveness to systemic chemotherapy and/or biologic agents. In addition, the decision to treat such a unique and variable demographic range of patients will likely depend on age, predicted survival, rapidity of disease progression, morbidity of procedure, capacity to recover, and patient preference. It is in this arena that SBRT can provide support to the surgical management of these patients due to its efficacy and lack of serious side effects.

Nevertheless, there are several limitations to our single institutional study, including the small sample size, tumor and biologic heterogeneity, dose variation, and selection of patients, all of which were unavoidable when evaluating such a subset of a given population. Although we report an excellent overall survival, this is more likely secondary to the specific biology of the disease and appropriate patient selection. In our population, selection bias may have occurred in opposing directions where SBRT was indicated due to their favorable small size and/or multiple locations, and in other times where surgery is unlikely due to the larger size of a lesion that may have been too central, requiring a pneumonectomy.

The reported safety and efficacy of treating LM are consistent in the literature, extrapolated from the much more extensive experience of treating metastatic disease in the lung from other primaries. Dating back to 2006, the University of Chicago group had reported a 3-year actuarial control rate of 82.5% out of 125 lung lesions treated from various primary malignancies [30]. SBRT may also be especially suitable for managing lung metastases because of the lack of concern for lymph node metastases and the fact that many sarcoma patients may have preserved lung function as opposed to primary lung cancer patients typically complicated by smoking and chronic obstructive pulmonary disease.

## 5. Conclusions

Our single-institutional experience suggests that SBRT for pulmonary metastases from HGS provides excellent local control with minimal toxicity. As our ability to radiographically detect small areas of disease increases, the practical efficacy of high-dose SBRT can be advantageous as it is an attractive less-invasive alternative for patients with high operative risk. Durable local control of pulmonary metastases can preserve the quality of life and likely also translate into an overall survival advantage in the same way the standard of care, metastasectomy, has demonstrated. The lack of hospitalization, efficiency, and safety of SBRT makes it an especially suitable back-up to surgery especially as the probability of a complication outweighs the potential benefit in high-risk or previously operated patients.

In the context of adjuvant chemotherapy and molecularly targeted agents, SBRT can work along with metastasectomy to provide long-term survival and prevent/alleviate symptoms in patients with metastatic HGS to the lungs. Furthermore, larger prospective studies need to be completed to determine specific selection criteria for the utilization of SBRT for HGS patients with LM. Also, modern techniques such as molecular profiling may provide insight as which patients are likely to have lung only metastatic disease, and thus benefit from local therapy. At our institution, a phase II feasibility study is planned to prospectively validate our findings.

## Figures and Tables

**Table 1:** Patient and tumor characteristics.

Age at time of treatment (years)	
Median	56
Range	12–85
Patients (*n* = 16)	
Male	7 (44%)
Female	9 (56%)
Lesions (*n* = 25)	
Peripheral	19 (76%)
Central	6 (24%)
Histology	
Leiomyosarcoma	7
Synovial cell	5
Osteosarcoma	4
Liposarcoma	2
NOS	2
Spindle cell	1
Chondrosarcoma	1
Liposarcoma	1
Hemangiopericytoma	1
Embryonal	1
Dose fractionation and BED*	
54 Gy, 3 fractions (BED = 151.2 Gy)	13 (52%)
50 Gy, 4 fractions (BED = 112.5 Gy)	9 (36%)
36 Gy, 3 fractions (BED = 79.2 Gy)	2 (8%)
42 Gy, 3 fractions (BED = 100.8 Gy)	1 (4%)

*Biological equivalent dose is calculated per (1), assuming *α*/*β* ratio of 10 for tumor (*n*  = number of fractions, *d* = total dose).

**Table 2:** Adverse events (CTC, version 3.0): pneumonitis.

Grade 0	1 (4%)
Grade 1	24 (96%)
Grades 2–4	0
